# Is It Because You Don’t Want to? A Content Analysis of Police Executive Leaders’ Perceptions of Policewomen’s Careers in Europe

**DOI:** 10.3389/fpsyg.2021.713696

**Published:** 2021-09-27

**Authors:** Concha Antón Rubio, Merlin Patricia Grueso Hinestroza, Montserrat Marín López

**Affiliations:** ^1^Department of Social Psychology and Anthropology, University of Salamanca, Salamanca, Spain; ^2^School of Management and Business, Universidad del Rosario, Bogotá, Colombia; ^3^Spanish National Police, Palencia, Spain

**Keywords:** gender equality, police forces careers, perceptions of male and female, career barriers, policewomen

## Abstract

The impediments and barriers that women face in entering and developing a police career have received relatively little attention from researchers. As of today in Europe, despite the slow progress, the 25% barrier to female representation has already been overcome in several countries. However, many areas remain closed to women within police organizations. In this context, research was conducted based on a content analysis of the perceptions of 56 police officers, 28 men and 28 women with considerable police experience, occupying executive leadership positions from a total of 26 European countries. Data was collected through a questionnaire composed of 23 open questions. The results show a considerable gap between the perceptions of male and female police executive leaders with regard to access, career development and workplace conditions faced by policepersons. According to the results, the mirage of equality, dominant in the view of male police officers, is a major barrier to achieving real equality, both horizontally and vertically. What implications these results have on the strategies that police organizations should follow to achieve the challenge of inclusion are discussed, and new ways of analysis are proposed.

## Introduction

The role of women in the police during the nineteenth century was essentially of an auxiliary nature, a kind of civilian who acted almost like a midwife in custody and support work in the search and custody of female suspects, or in administrative tasks of aid and support (Martin, 1989; Appier, 1992; Schulz, 1993; Premier, 1998). Women were not really accepted or integrated as police officers until a few decades ago, and it can be said that today not only are they still a minority, but their representation, especially in management positions, is much lower (Natarajan, 2008; Cordner and Cordner, 2011; Barratt et al., 2014; Silvestri and Tong, 2020), although it is difficult to determine the number of women and their representativeness in different positions in European police organizations due to the lack of complete official statistics or other information resources (Brown, 2000).

To find out about the representation of women in the police force, objective data from EUROSTAT (2019) is used, which provides global data on men and women in police forces without making any reference to percentages disaggregated by categories or ranks. The analysis of global data obtained by EUROSTAT (2019), offers a positive evolution of the presence of women in the European police, although very different among European countries, which in 2008 was 16.7%, a figure that has increased to 21.28% in 2017. At present, the average is one woman per five men in Europe, starting from an average percentage of 318 police officers per 100,000 inhabitants, i.e., one police officer per 314 people. Of the 41 territories for which data are available, only 10 countries–Cyprus, England and Wales, Latvia, Lithuania, and Netherlands, Northern Ireland, Norway, Serbia, Scotland, and Sweden–reach or exceed 25% female representation.

Despite the general increase in the percentages of women in the police forces analyzed, the data cannot be interpreted uniformly. While it is true that a greater percentage of women in countries that have maintained or increased their number of personnel may respond to an improvement in the gender equity strategies followed by their organizations, when this percentage increase occurs in parallel with the reduction of the total police force, it cannot be said that there has been the aforementioned improvement in strategy. In these organizations, for example, it could be due to the retirement of the more veteran policemen who joined when the presence of women was not contemplated.

Based on the data from EUROSTAT (2019), there is no relationship between the year of entry of women into the respective police forces and their percentage representation in the various scales and categories. To this end, it should be borne in mind that in some countries, especially those with a military structure, the entry of women has been later on, so it is logical to expect that there are not high percentages in the police force. In other countries, mainly those that achieved independence, mainly former Soviet republics, there is a high percentage of entry compared to others that have had more years of democratic experience.

Moreover, equality measures or policies established in international and European standards give countries the option to determine their priorities, leading to their highly variable implementation across nations. Some countries have implemented a system of quotas, specific regulations, equality offices and official systems of mentoring and networking, as opposed to others that only seem to have general normative regulations that apply to all administrations. According to Prenzler and Hayes (2000) and Prenzler et al. (2010) the type of strategy is likely to have a major impact on the resulting gender equity.

For the time being, European police organizations are still far from the integration predicted by Brown’s (1997) model which held that women’s integration would be gradual and that it was only a matter of time before the differences would disappear–when women reached percentages close to 25%. Although this ratio has already been achieved in some countries, the results show that policewomen still consider there is still no real equality between men and women in European police forces (Archbold et al., 2010; Van Ewijk, 2011; EUROPOL, 2013).

Despite legal obligations, social justice requirements (Antón and Quesada, 2008; McMurray et al., 2010; Hughes, 2011), and evidence of the effectiveness of inclusive organizations (Luijters et al., 2008; Jansen et al., 2015; Grueso and Antón, 2020), the rate of change toward full inclusion of women in police organizations remains alarmingly slow having various types of undesirable impact such as the perception of isolation, loss of identity, low self-esteem and low self-efficacy, stress, low job satisfaction and lack of affective and continuance commitment (Kanter, 1977; Krimmel and Gormley, 2003; Stroshine and Brandl, 2011; Guillaume et al., 2012).

The literature review has shown that in police work there are barriers (Natarajan, 2008; Silvestri and Tong, 2020), prejudices and stereotypes (Natarajan, 2008; Archbold et al., 2010), and culture manifestations (Dick and Jankowicz, 2001; Barratt et al., 2014), that hinder the incorporation and career advancement of women (Silvestri and Tong, 2020). Given that leaders in organizations play a role in overcoming or perpetuating such organizational dynamics the following questions guide our study: (1) From police executive leader’s perception, how desirable is to develop measures to achieve gender parity in the staffing structures? (2) Do they perceive that women differ from men in policing? (3) How they perceive the culture, organizational structure and equality policies? (4) Are there differences between men and women executive leaders?

With the understanding that police organizations have some shared characteristics with other typically male organizations, our research advances the understanding of the perceptions that female and male police officers have of the incorporation and career development of women police. To this end, we begin with a review of the main topic, discussing the incorporation and career development of female police officers. Secondly, we develop the research method, followed by the results, discussion and finally conclusions and study limitations.

## Theoretical Framework: Barriers and Controversies Over Female Police Officers in the Workplace

According to O’Connor et al. (2011) gender discrimination is what has slowed women’s career development the most in certain professions that, both historically and culturally, have been regarded as masculine, such as the police or the military. It is clear that police work imposes physical and emotional demands. As well as that, such work is strongly associated with masculine traits of power, authority, strength and protection, so that from the beginning the incorporation of female police officers has been a subject of controversy (Martin and Jurik, 2007). According to Natarajan (2008) there are three barriers that have hindered the incorporation of women into police organizations: (a) long-standing socio-cultural perceptions of the nature of police work; (b) features of the organizational structure of law enforcement and the police subculture; and (c) pervasive stereotyping of female police officers by their male colleagues, supervisors, and the public at large.

Historically, the role of the policeman has been played by men and the result is that, not only has their effectiveness or capabilities not been questioned, but the erroneous conclusion, never empirically endorsed, has been reached that the qualities required to be a good policeman are those that are stereotypical of male culture exclusively (Lanier, 1996; Archbold et al., 2010; Van Laar et al., 2017). Thus, despite the gradual integration of women into the police forces, first by carrying out auxiliary tasks and, progressively, in more operational tasks, it may be that the career development of women is mediated by formal and informal barriers and obstacles linked to gender and organizational culture (Silvestri and Tong, 2020).

Based on the factors proposed by Natarajan (2008) and Silvestri and Tong (2020) regarding the differentiated conditions faced by men and women in the workplaces, the literature review is developed as follow: (a) socio-cultural perceptions of the nature of police work: demands of the post, compatibility between the professional and personal role; (b) organizational structure and the police subculture: police subculture and discrimination; and (c) Stereotyping of female police work: motivations, specific capabilities.

### Socio-Cultural Perceptions of the Nature of Police Work

The nature of policing has historically excluded women from its ranks mainly due to perceptions of policing as stressful, dangerous, physically demanding, and a sexist occupation (Diaz and Nuño, 2021). The literature review identifies two major themes surrounding the nature of police work, as described below.

#### Demands of the Post: Physical Competence and Soft Skills

Despite studies to the contrary, there are strongly rooted clichés in police culture about women’s physical competence to cope with police work.

According to Rabe-Hemp (2008), a central object of controversy is that women do not possess sufficient physical strength, which limits them in certain operational functions, especially those involving the use of physical force. Women have a build, a size, an appearance, which is associated with weakness, and may be easier to attack or be perceived as less authoritative. Despite being a recurring argument, it is curious that it is mostly absent when supervisors are asked about the skills required to perform police tasks (Brown et al., 1993).

Several authors state that being a good police officer does not depend on physical strength or dominance, but on the ability to think and act with human sensitivity and interpersonal skills–the soft skills–(Lanier, 1996; Natarajan, 2008). Studies conclude that women are considered at least as equal as men in most areas of police work (Poole and Pogrebin, 1988; Price, 1996; Breci, 1997), that they are as effective as men in patrol car tasks (Martin, 1989), that there are no consistent differences in their field performance (Worden, 1993), and that females are no less involved and no less able to meet the physical requirements of police work (French and Waugh, 1998). Therefore, it has been demonstrated that gender is not a valid reason for discriminating against street work.

On the other hand, Bartol et al. (1992) argue that although the sources of stress are basically the same for men and women, the latter are more vulnerable to stressors linked to tragedies, safety of the public and their professional colleagues. However, He et al. (2002) study indicates that women have better coping mechanisms for depression.

#### Compatibility Between Family and Professional Role

According to various studies (Eagly et al., 2003; Van Engen and Willemsen, 2004; Antón, 2006; Eagly and Carli, 2007) one of the main barriers arises from the duality of family vs. work. In this way, the reality is that many women perceive that they must choose between home and work, for which reason they tend to abandon their development and progress in the organization, due to the incompatibility of adequately fulfilling the roles of being a good professional and being a good mother at the same time (Muller et al., 2020).

A deeply rooted socio-cultural perception about police work is that it requires long working hours, availability at weekends and nights, as a model of the value of sacrifice for organization, all of which seem to demonstrate more commitment, although such efforts are neither necessary nor rational (Rapoport et al., 2002; Brett and Stroh, 2003). Silvestri (2013) suggested that police organizations have created an environment adjusted to men’s preferences and the assumption of a male lifestyle. This is what has been considered valid, exemplary and recognized and, in some way, excludes women in human resources policies, with erroneous beliefs that they do not fit the profile and they will abandon or have greater absenteeism, or because the cost of pregnancies and maternity (Dick and Jankowicz, 2001).

In the police, there is this stereotypical belief that “mothers care and leaders assume responsibility,” which perpetuates the belief of role conflict: women, at certain times in their lives, experience contradictory feelings about what are their goals and priority tasks, feelings that do not appear so frequently in men, who do not usually face this dilemma or are expected to do so. Mainiero and Sullivan (2005) in their study concluded that men within organizations kept their personal lives and work separate and that they could do so because their partners normally dealt with the delicate game of balancing work and personal issues.

### Organizational Structure and the Police Subculture: Cultural Manifestations in Police Institutions and Discrimination

According to different authors, policing is a male-dominated profession that perpetuates the wrong idea that women do not belong on the force and the efforts to gain equality within policing are often unsuccessful (Diaz and Nuño, 2021). Situations of inequity, on the other hand, may be related to the different assimilation strategies followed by police institutions (Heidensohn, 1998).

#### Cultural Manifestations in Police Institutions

One of the recurring themes to explain the under-representation of women in the police force is organizational culture (Dick and Jankowicz, 2001; Barratt et al., 2014). The general idea is that the organizational culture of the police force promotes masculine values and reward men for displaying male traits and behaviors. This orientation has been termed “police subculture” (Natarajan, 2001). As Sahgal (2007) states, although women have experience and are qualified, the predominance of men has resulted in organizational culture being governed by patterns of behavior that are perceived as the unquestionable norm, so women have often found it difficult to be accepted as equals by their peers. In addition, women are generally not assigned important roles or the opportunity to handle challenging jobs, which deprives them of recognition and power-sharing. In mostly male organizations, such as the police, dissimilarities lead to salient stereotypically negative and devalued social identities, which do not affect women and men equally (Archbold et al., 2010).

One manifestation of organizational culture is related to the establishment of informal communication networks. Evidence suggests that women, including police officers, do not frequent certain “masculine” places, where relevant information can be generated and important decisions can be made, so it is to be assumed that such decision-making, subtly, underhandedly, or even unconsciously, causes men to support other men to move up the power hierarchy in a “homosocial reproduction” process (Kanter, 1977; Bass and Avolio, 1994). The members of the police forces have been mostly male, so the leaders are male, and they are the ones who finally make the strategic decisions as well as the selection and promotion of personnel. Several studies show that gender stereotypes also exist in relation to career development.

Ridgeway (2002) argues that the behaviors of men and women vary by virtue of the type of organizational culture, the type of tasks to be performed and the sexual composition of employees. Regarding to the organizational culture of the police institution, women seem to approve much less of the organizational culture of policing than men, and although they try to accommodate themselves, in many cases they perceive that they cannot show their own identities or preferences, and it only remains for them, like more and more men who do not fit into that culture, to invent multiple ways to advance while trying to change the culture so that it is more inclusive in values, needs and priorities (Eagly, 2007; Van Vianen and Fischer, 2010). Thus, organizational culture has an impact on the way women behave.

In general, the dilemma for women, with regards to social identity, is the question of whether they should behave as “POLICEwomen (defeminized) or PoliceWOMEN (Deprofessionalized)” (Martin, 1979, p.134). If they behave like women–policeWOMEN–they suffer a low acceptance on the part of their companions, and if they behave like men–POLICEwomen–they tend to run the risk of losing the respect of their peers (Suman, 2010). This situation increases their stress at work, due to the ambiguity of their role (Jacobs and Schain, 2009).

The drawbacks of undoing gender (Butler, 2004) refer not only to one’s own self-concept, but to the erroneous perception that by adopting masculine roles and thus undoing gender, a woman’s success can be attributed precisely to her having given up her already devalued feminine gender. In this sense (Hoobler et al., 2011) it has been stated that men judge unethically and unfairly women who acquire success by adopting masculine roles, considering that they violate gender roles. In addition, women who undo their gender in order to adapt to the masculine majority, thus, establish an absence of feminine leadership role models, do not change the organizational culture and maintain gender stereotypes.

Police subculture also has an impact on how leadership styles are valued depending on whether the leader is a man or a woman. The role incongruence theory proposed by Eagly and Karau (2002) argues that people tend to believe that to occupy and effectively perform leadership positions it is necessary to display masculine qualities. Österlind and Haake (2010) state that, within police organizations, women are under-represented at the leadership level because they tend to exhibit characteristics considered too soft and ineffective and characteristic of a people-oriented leadership or “feminine” style of leadership (Rosener, 1995; Jacobs and Schain, 2009). As Butler (2004) states, this has caused women in male organizations, such as the police, as they have gained leadership positions, to deploy leadership styles similar to men, in order to reduce the incongruence between being a leader and being a woman, since in reality, according to the descriptive and prescriptive components of stereotypes. Therefore, when women present their ideas in an assertive and managerial manner (the prescriptive role is broken), they are often perceived as unreliable, cold and incapable of exerting influence and people tend to react more negatively toward them than toward men with similar characteristics.

Foschi and Lapointe (2002) affirm that women leaders can be evaluated negatively for two reasons, firstly because they display characteristics different from those considered effective for leadership, or secondly because if they are displayed they are not very feminine. Thus, in both cases, there is incongruence between the leadership role and the social role. This powerful inhibitor, the “Behavioral Double Bind” (Gerber, 2001; Berbel, 2014; Hopkings and O’Neil, 2015), supposes that women suffer on the one hand the expectation that as leaders they must be abrupt and authoritarian in order to gain respect, but upon doing so are cataloged as aggressive or hysterical. On the other hand, if they are shown to be female, they run the risk of appearing incompetent as they do not fit the male model of competition. In this context, negative attitudes force women to make a greater effort to show their worth because they face very rigorous judgments while, in the case of men, much clearer and stronger evidence of poor performance is required for them to receive a negative evaluation.

In this way, women, as they ascend to higher hierarchical positions, not only must make a greater effort to break the incongruence by displaying different characteristics associated with the type of leadership that is considered effective, or characteristics that are too feminine and therefore not valued, but also, the tendency toward prejudice implies that they must be less visible, make fewer mistakes and settle for second-level positions, of little value to men, or assume tasks condemned to failure (Foschi and Lapointe, 2002).

However, there is also evidence of the positive impact on career development of masculinity for women in police organizations, both because they display behaviors congruent with the “appropriate” leadership role and because their acceptance allows the ideal of masculinity to be perpetuated in these organizations (Acker, 1992; Schippers, 2007). In this way, the studies in police environments are congruent with those obtained in other environments, in which it has been shown that women are really successful in previously male worlds and positions, especially at a high level, when they are perceived with fewer stereotyped attributes of women and more attributes of men and roles of male bosses (Schein, 2002; Eagly and Carli, 2007; Heilman and Okimoto, 2007; Derks et al., 2016) although it is possible that this relationship may vary according to their sexual orientation (Barratt et al., 2014).

#### Discrimination

Heidensohn (1998) argues that there are two models for integrating women into the police, the “gendered model,” which reserves some positions within the organization for women, for which they are considered especially qualified, and the “integration model,” which is the result of equal opportunity regulations. Despite the laws, the integration model does not guarantee automatic equality between men and women. According to Brown’s (1997) proposal, from the time that legislation obliges the recognition of women’s rights to enter and obtain any position available in the police, until this becomes a reality, police organizations go through different phases characterized by the lack of equity between men and women.

Brown (1997) argues that once the initial resistance to the incorporation of women is overcome, they will have to face successive situations of sexual harassment–*take-off stage*–discrediting and backlash campaigns–*stage reform*–and, finally, glass-ceiling–*tip over*.

Faced with this situation, Brooks (1997) consider that an internal barrier was the lack of understanding of the organizational culture, so for a woman it was important to learn that success was to work harder than her male colleagues and to assimilate that discrimination is based on fear of the unfamiliar or unknown.

Although the existence of a male police culture has also been linked to other forms of discrimination, such as sexual harassment, or dirty jokes reported in several investigations (Shadmi, 1993; Martin, 1996), the issue that seems to be most pronounced today is the underrepresentation of women in leadership positions in police organizations–the glass ceiling.

Some authors argue that the tendency toward prejudice and discrimination becomes greater as one moves up the hierarchy, so men are believed to be more effective in first-level positions while women are more effective in second-level positions (Eagly et al., 1995; Paustian-Underdahl et al., 2014).

### Stereotyping of Female Police Work: Specific Capabilities and Motivations

Segregation in the police, both vertical and horizontal, is a fact (Blackburn and Jarman, 2006). For some authors, segregation is the result of the existence of specific needs within police work (i.e., dealing with women and children) and of the different interests and qualities of men and women. From positions close to the gendered model, it is argued that while men tend to choose more masculine environments, women are more attracted to positions where the tendency is to collaborate and not to control others. For example Silvestri (2013) explains that women may seem better in middle ranks, where interpersonal skills are more frequent and where they feel more accepted and similar.

#### Specific Capabilities

The existence of specific skills linked to women that would enable them to perform better in certain areas of policing has been defended by various authors as the best guarantee of achieving the same status as men in the police force (Natarajan, 2008). Kakar (2002), for example, argues that women are socialized to be mothers or caregivers, assistants, and that they are passive, although they have good communication skills, so they would be suitable for certain situations or police specialties, related to providing assistance to victims and being present in situations where soft skills are needed to interact.

What seems to be demonstrated, continues Kakar, is that precisely “those soft,” devalued competences “are an advantage in certain situations, since women, are more difficult to anger, tend to take more time, have more patience, tend to delay more in the use of force, are proactive negotiators to try to reduce the contextual tension, which is sometimes considered a weakness according to other currents, since they can lower the guard trying to be empathic and are easier to attack” (2002, p.241).

Lonsway et al. (2003) state that women engage in less conduct associated with police brutality, disciplinary infractions and, above all, they engage in less sexual harassment or mobbing conduct, so the advantages that the presence of women bring to police organizations, as well as the specific skills they possess, must be taken into account.

Female leadership has been related to Transformational Leadership, which is considered one of the most effective styles today (Bass and Avolio, 1994). Various studies (Carless, 1998; Ramos et al., 2003; Hernández et al., 2014) conclude that women tend to develop this style of leadership more. In police organizations, the acceptance of the greater effectiveness of transformational leadership has created a certain dilemma since the passage from an autocratic leadership (of control) characteristic of the military and police tradition, and the transactional leadership (transaction between those who decide the objectives and the means to achieve them and those who must execute the entrusted orders), characteristic of the masculine stereotype, toward a transformational leadership, would suppose the acceptance of the so-called soft competences and therefore of an increase of women in command positions (Kingshott, 2013).

Women have repeatedly been shown to be successful at implementing this leadership style and multiple studies have shown that are able to bring about effective organizational change, two skills that are both necessary and desired in police services (Ayman et al., 2009; Vinkenburg et al., 2011).

#### Motivation

Another controversial issue relates to the preferences and commitment shown by women police officers.

Although there is evidence of important overlaps between the occupational preferences of men and women (Holdaway and Parker, 1998), it is also argued that women tend to flee from operational assignments and tasks and prefer to take office positions, while others authors state that women do not reject such positions, but approach work from a different perspective than men, and that it is sometimes their feeling of isolation and non-integration that causes them to leave positions in which they feel continually analyzed, being a minority (Kanter, 1977; Grimm, 2010).

Morash and Haarr (2012), conducted a qualitative study of female police officers’ identities, and concluded that they identified much more with occupational identities than with stereotypical gender identities, preferring to form deep alliances in which they could be themselves, rather than superficial networks and, that their essential motivations for advancement included greater opportunities to contribute to or impact on the organization, personal satisfaction to improve and deliver, greater challenges and responsibility and greater control over projects, people and decision making, while those of men were aimed more at gaining power and authority. These ambitions and strategies have also been reported in research on women police in some traditional societies (Natarajan, 2001).

It is also common to believe that women do not want to be promoted (Dick and Jankowicz, 2001), or that they abandon their career purpose for lack of motivation to achieve, but it is possible that women, in reality, suffer role overload (increase in tasks that women perform by combining several roles), their work is doubled and sometimes tripled when they must manage the cultural and social role assigned to household tasks and assistance to children or parents (Holdaway and Parker, 1998).

The world has changed, and the old-fashioned view of law enforcemetn as a physical and aggressive profession where only men can function is no longer valid (Hughes, 2011). The new concept of public service of the police forces, which are moving toward community police budgets, must integrate, together with this reactive character of control, a new proactive culture, in which the characteristics, not only physical but also psychological, and effective communication are essential (Loftus, 2010).

On the other hand, research shows that the perception of a change of positive diversity is related to a greater organizational commitment, identification and, therefore, greater effectiveness and less possibility of abandonment, and that diversity and gender equity are the best predictors of legitimizing the advancement of women to positions of leadership since the change of criteria and values within organizations (Luijters et al., 2008; Jansen et al., 2015). Despite this, European police organizations are not achieving the expected and enforceable women’s participation at the moment.

Given the low presence of women in police organizations, it is not possible to determine whether organizational commitment to gender mainstreaming is merely rhetorical and derived from compliance with legal obligations. Advocating for the integration of women into institutions could “trigger resistance to gender mainstreaming when individuals within an institution internalize the existing informal unequal gender norms and might act to preserve the *status quo*” (Mergaert and Lombardo, 2014, p. 6).

Individual resistance to organizational change–such as gender mainstreaming–whether explicit or implicit, cannot be underestimated (Benschop and Verloo, 2006). The experience of the authors of this research as trainers in gender mainstream courses in police organizations coincides with that of Lombardo and Mergaert (2013) in finding that there are three explicit areas of resistance: “denial of the need for gender change,” “trivialization of gender equality,” and “refusal to accept responsibility for solving the problem.”

The review of the literature has revealed the existence of controversies and phenomena linked to the incorporation and leadership of women in police organizations that have yet to be resolved. Moreover, gender mainstreaming in the police is a relevant organizational change that requires the unwavering support of organizational leaders. The scarcity of investigations carried out in police organizations, particularly in Europe, and the scarce consideration of the perceptions of all officers, make their study relevant (Natarajan, 2008).

Therefore, the research carried out here has the purpose of getting to know the perceptions of police men and women on the incorporation of women in the Police, their promotion and career development, as well as the potential barriers they may suffer, the impact of different cultures, structures and equality policies, with the aim of suggesting strategies to overcome the situation of inequality in the current percentage of representation.

## Research Method

### Type of Study

To develop the research, a qualitative approach was used.

### Sample and Data Collection

The study survey sample from a total of 26 European countries was comprised of 56 experienced police officers in executive leadership positions, of which 28 were men and 28 were women. The choice of police executive leaders sought to ensure that the participants had a broad vision of the organization with a relevant role in the implementation of the gender perspective. The inclusion of more than two participants per country is justified by the existence of different types of police organizations in the country and for reasons of opportunity. Two police officers from each of the countries included in Table 1 participated, as well as the responses of four women and four men from Romania and Spain, respectively. The police experience of the participants ranged from 7 to 41 years, all having leadership roles at the time of completing the questionnaire.

**TABLE 1:** List of countries, police forces’ characteristics and respondents.

Country	% Females (1)	Police Structure (2)	Equality Regulation	Equality Office	Quots	Mentoring/Networks	Gender (3)	Experience	Rank
AT AUSTRIA	20%	M	Yes	Yes	Yes	Yes	F	16	Inspector
							M	18	Senior Manager
BE BELGIUM	22.7%	C	No	Yes	No	Yes	F	12	Chief Inspector
							M	28	Superintendent
BG BULGARIA	11.2%	C	Yes	Yes	No	No	F	10	Chief inspector
							M	18	Commissioner
CZ CZECHIA	15.4%	C	Yes	Yes	No	No	F	20	Lieutenant Colonel
							M	23	Colonel
CY CYPRUS	25.4%	C	Yes	Yes	No	No	F	23	Inspector
							M	28	Superintendent B’
DK DENMARK	15.6%	C	Yes	No	No	No	F	14	Inspector
							M	16	Inspector
DE GERMANY	21.9%	C	Yes	Yes	No	Yes	F	36	Senior detective inspector
							M	41	Chief superintendent
EE ESTONIA	35.2%	C	Yes	No	No	Yes	F	33	Lieutenant Colonel
							M	20	Lieutenant Colonel
IE IRELAND	33.4%	C	Yes	Yes	No	No	F	20	Superintendent
							M	26	Sergeant
EL GREECE	13.7%	M	No	No	No	Yes	F	22	Lieutenant Colonel
							M	9	Captain
ES SPAIN	11.3%	M	Yes	Yes	No	No	F	17	Major
							M	31	Lieutenant Colonel
		C	Yes	Yes	Yes	No	F	23	Commissioner
							M	25	Commissioner
FR FRANCE	19.2%	C	Yes	Yes	No	Yes	F	16	Commissioner
		M	Yes	Yes	No	Yes	M	19	Lieutenant Colonel
HR CROATIA	18%	C	Yes	No	No	Yes	F	22	Inspector
							M	22	Advisor
IT ITALY	8.5%	M	No	No	No	No	F	11	Captain
							M	25	Colonel
LV LATVIA	58.3%	M	No	No	No	No	F	15	Lieutenant Colonel
							M	16	Lieutenant Colonel
LT LITHUANIA	38.6%	C	Yes	No	No	No	F	20	Commissioner Inspector
							M	23	Lieutenant Colonel
HU HUNGARY	22.8%	M	Yes	No	No	No	F	20	Major
							M	25	Lieutenant Colonel
NL NETHERLANDS	32.8%	C	Yes	Yes	Yes	Yes	F	34	Commissioner
							M	28	Chief Inspector
PL POLAND	16.0%	M	Yes	No	No	No	F	21	Lieutenant Coronel
							M	19	Lieutenant Coronel
PT PORTUGAL	7.8%	C	Yes	No	No	No	F	19	Inspector
							M	21	Major
RO ROMANIA	18.7%	C	No	No	No	No	F	23	Chief Commissioner
							F	11	Sub-Commissioner
							M	32	Colonel
							M	17	Chief Commissioner
SK SLOVAKIA	18.1%	C	Yes	No	No	No	F	7	Captain
							M	16	Lieutenant
FI FINLAND	17.8%	C	Yes	No	No	No	F	20	Chief Inspector
							M	28	Superintendent
SE SWEDEN	32%	C	Yes	Yes	No	Yes	F	15	Inspector
							M	20	Superintendent
UKC-L ENGLAND AND WALES	41%	C	Yes	Yes	No	Yes	F	18	Chief Inspector
							M	17	Superintendent
RS SERBIA	30.2%	C	Yes	No	No	No	F	18	Advisor
							M	20	Major

*(1) According to the latest data available at Eurostat in February 2020 (Source crim_just_job).*

*(2) M = Military; C = Civilian.*

*(3) M = Male; F = Female.*

Participants in the study were selected on the basis of a purposive sampling in the course of Future Leaders of European Union Agency for law Enforcement Training (CEPOL), in the European Joint Master Programme of Cepol (EJMP), in the European Joint Master’s in Strategic Border Management Programme (EJMSBG) of the European Border and Coast Guard agency (FRONTEX), as well as through snowball sampling contacts in several European agencies such as The European Union Agency for Law Enforcement Cooperation (EUROPOL) and The Center for the Democratic Control of Armed Forces (DCAF). In this regard, the participants were elected because they met the criteria predetermined by the researchers.

Of those invited, only two people refused to participate because they could not get permission to do so from their organizations. The countries that did participate are: Austria, Belgium, Bulgaria, Czenia, Cyprus, Denmark, Germany, Estonia, Ireland, Greece, Spain, France, Croatia, Italy, Latvia, Lithuania, Hungary, Netherlands, Poland, Portugal, Romania, Slovakia, Finland, Sweden, England and Wales, and Serbia, as seen in Table 1.

All participants in the study, in compliance with ethical requirements, have been informed that the use of their answers is restricted to the scientific field, they have been guaranteed anonymity and the right to be informed of the results once the analysis of the data has been concluded. Given the sensitive nature of the information provided, the participants were specifically guaranteed that the results would not include their age or specific position, so that they would not be identified.

### Data Collection

A qualitative data collection was carried out after a formal request to the participants, who have formalized these questionnaires on line, English being the language used.

In the process of elaborating the questions for the questionnaire, the contributions made in the scientific literature of instruments designed to value perceptions was taken into account. In addition, four civilians from Sweden, Denmark, United Kingdom, and Greece, two men and two women, contributed by peer reviewing the contents and to the process of elaboration of the questionnaires.

The 23 open questions included in the questionnaire (see Supplementary Annex 1), plus the demographic data necessary for the study were designed for the collection and subsequent analysis of the different perceptions of men and women toward the main controversies related with the entry and career development of women in European police organizations. Each question incorporated into the interview guide contributes to the resolution of the general research questions, as indicated in Supplementary Annex 1.

The questionnaires received were subjected to quality control, and those that did not provide answers to some questions in the questionnaire were eliminated from the analysis, taking into account the criteria established to evaluate the internal and external validity and reliability set by Tracy (2010). The ten incomplete questionnaires were used for the intercoder agreement analysis. No relevant patterns were found with respect to unanswered questions.

### Techniques for Data Analysis

In this research a Content Analysis (qualitative for the first phase and quantitative for the second phase) was used as analytic strategy. During the first phase Qualitative Content Analysis–QCA–was used. According to Hsieh and Shannon (2005), QCA can be developed in three ways: conventional, directed, and summative content analysis. In this research we used a conventional content analysis which is described as inductive category development generally used when the researchers aims to describe a phenomenon (Hsieh and Shannon, 2005). In conventional content analysis, the researchers allow the categories and names for categories to flow from the data (Hsieh and Shannon, 2005).

The Data analysis procedure started by repeatedly reading all data from the interviews to achieve immersion and obtain a sense of the whole. Afterward, data was read word by word to derive first order codes. As this process continues, labels for first order codes emerged and definitions for each first order code were developed (Table 2). Then, first order codes were sorted into categories based on how different codes were related and linked. These emergent categories were used to organize and group codes into meaningful clusters–second order codes. Next, definitions for each second order code were developed (Table 2).

**TABLE 2:** First and second order codes.

First order codes*	Operational definition	Second order codes	Operational definition
Operational/frontline sub-representation	It is expressed that women are underrepresented in operative or frontline tasks.	Perceived adequate representation	Referring to the perception of the number of women belonging to the police in each country.
Special unit sub-representation	It is considered that women are underrepresented in special units.		
Command and control sub-representation	It is affirmed that women are underrepresented in command or control positions.		
Need for policewomen role models	It is said that quotas are necessary because they generate models for policewomen.	Supporting female quotas	Referring to the opinion that participants have about the necessity of gender quotas in their respective police organization.
Equality in practice	It is defended that quotas guarantee parity of male and female representation in the staff.		
Networking	It is affirmed that the existence of a minimum number of women to be able to create networks is necessary.		
Discriminatory to men	It is said that the existence of quotas for women is discriminatory toward men.		
Resistance to change	It is maintained that the politics of quotas are inefficient because they increase the resistance to change.		
Reverse impact: Perception of women’s lack of merit.	It is affirmed that the existence of quotas can lead to the questioning of the merits of the women who obtain these positons.		
Soft (values, social skills, emotional control)	Social abilities, emotional control and or values are mentioned as relevant competences in police work.	Police relevant Competences	Referring to the perception held of the competences necessary to appropriately carry out the role of a police officer.
Hard	The technical competencies and or the knowledge of the laws and operational protocols are mentioned as relevant.		
Physical condition	It is said that police work requires strength, stamina, physical appearance or other factors related to physical condition.	Physical strength required	Referring to the participants’ perception of the physical capacity required to appropriately carry out the role of a police officer.
Choice of shifts	It is affirmed that family responsibilities determine the choice of shift.	Work-life balance	Referring to the participants’ perception of the impact of family responsibilities on workplace decisions and performance.
Choice of jobs	It is maintained that family responsibilities condition the choice of the type of work position.		
Time off work and leave	It is said that family responsibilities imply time off work and or the taking of leave.		
Performance	It is said that family responsibilities have a negative impact on work performance.		
Reproduction of traditional values	It is mentioned that organizational culture reproduces traditional social values.	Male police subculture	Participants’ perception of the characteristics of the police organization in terms of its occupational culture.
Power and decision-making structures	It is said that police culture excludes women from positions of power and the structure of decision making.		
Promotion of masculinity	It is maintained that the competencies valued by the organizational culture are those considered to be masculine and or directly to being a man.		
Devaluation of women	It is affirmed that the organizational culture shares negative stereotypes of women.		
Devaluation of female heads	It is said that the organizational culture devalues female leadership styles.		
Design of working hours	It is expressed that organizational culture demands dedication to work via a long working day.		
“Victims” of culture	Different groups of workers (women, non-traditional men, homosexuals) are considered prejudicial for the organizational culture.		
Old school vs. present day	The existence of a masculine police culture is denied even though it is accepted that it existed in the past.		
Reflection of society	It is expressed that organizational culture is simply a reflection of society so it is impossible to talk of a male police subculture.		
Horizontal discrimination	The existence of impediments and barriers for women to access positions in specific police units.	Perceived discrimination	Referring to participants’ perception of the existence of specific discrimination directed toward women.
Vertical discrimination	The existence of a glass ceiling for women.		
Wage discrimination	Salary differences that prejudice women are shown.		
Psychological harassment	The existence of the psychological harassment of women based on gender alone.		
Isolation	It is cited that women are isolated in terms of work and or that men refuse to work with them.		
Scrutiny	The existence of a monitoring control system that penalizes the development of women above that received by men is mentioned.		
Denial of discrimination against women	It is expressly denied that women suffer more discrimination than men.		
Women’s values	Reference is made to the relevant values more present in women for police work than in men.	Female specific skills	Referring to participants’ perception of the study of the specific capacities of female police officers.
Women’s social skills	It is expressed that women possess social skills that make them more efficient in some work positions.		
Women’s emotional control	It is affirmed that women possess more emotional control than men.		
Women’s hard skills	It is expressed that women are more qualified professionally than men.		
Lack of commitment	It is affirmed that women do not really get involved in police work, not being willing to take on shift work or the weekly hourly load for some work positions.	Motivation	Referring to the participants’ perception of the level of career development motivation of female police officers.
Lack of ambition	It is maintained that women do not have promotional ambitions.		
Preference for the non-operational	It is affirmed that women feel more comfortable in administrative positions than operative ones.		
Vulnerability	The lack of physical strength to confront aggressors and critical situations is expressed.	Capacity and strength	Referring to the participants’ perception of the study of the adaption of women to the police role.
Biological specificities	Reference is made to female biological specifics (menstruation, pregnancy, micturition) as obstacles to police work.		
Hostile sexism	Distrust of the nature of women and their suspected tendency to cloud the working atmosphere.		
Selection Processes	The admissions system to the police organization is expressed to be a barrier for women.	Female specific organizational barriers	Referring to the perceived organizational barriers that affect women in a direct manner.
Assessment and recognition of merit	The system for the evaluation and recognition of merit is expressed to be a barrier.		
Promotion processes	The promotion process is expressed to be a barrier.		
Lack of support and networks	The lack of support and isolation within the organization are expressed to be barriers for women.		
Police subculture	The existence of cultural police stereotypes that prejudice women in their career development is referred to.		
Equal opportunities	It is affirmed that equal opportunities for men and women in the police exist.		

In the research second phase, a Quantitative Content Analysis was developed. Quantitative Content analysis is defined as “the intellectual process of categorizing qualitative textual data into clusters of similar entities, or conceptual categories, to identify consistent patterns and relationships between variables or themes” (Julien, 2008, p. 120). In this quantitative work, quantitative content analysis was applied producing frequencies based on preselected categories following the directions of Julien (2008). For this activity, the degree of agreement among researchers regarding to the second-order codes was calculated with the Krippendorff’s Alpha reliability coefficient (Table 3). Based on the second-order codes and the value labels assigned, the researchers proceeded to perform quantitative analyses.

**TABLE 3:** Second order codes and value labels.

Second Order Codes	Description	Value Labels	% agreement (Krippendorff’s Alpha)
Percentage of women in the organization	The percentage of women that the participant perceives to be in the organization is noted	numerical	
Perceived adequate representation	This category expresses whether the participant values the representation of women in leadership positions tasks and ranks.	1 = inadequate 2 = adequate	100% (1)
Supporting female quotas	Supporting female quotas into de organization	1 = pro quotas 2 = no pro quotas	90% (0.90)
Police relevant competences	This category examines the kind of skills are considered police relevant competences	1 = soft skills 2 = soft and hard skills	90% (0.90)
Physical strength	This category examines if physical strength is required in police work.	1 = required 2 = no required	100% (1)
Work-life balance	Participants are assessed on whether they perceive that work-life balance is possible in women’s police careers.	1 = yes 2 = no	90% (0.90)
Male police subculture	Codified when participants define police culture as (1) masculine culture, (2) masculine reflection of society, (3) masculine in the past	1 = male culture 2 = reflect of society 3 = old school	86% (0.86)
Perceived discrimination	The violence, harassment, and discrimination against women and men are compared	1 = bigger against women 2 = no differences	100% (1)
Female specific skills	This category expresses whether the participant considers that women have specific skills	1 = yes 2 = no	100% (1)
Motivation	This category reflects whether the participant considers that women lack the motivation to be good police officers	1 = yes 2 = no	90% (0.90)
Capacity and strength	This category assesses whether participants attribute less capacity and strength to women in police work.	1 = yes 2 = no	90% (0.90)
Female specific organizational barriers	The participant considers that women face specific organizational barriers in their career development	1 = yes 2 = no	100% (1)

### Procedure

The coder team was comprised of all three researchers, all experienced in qualitative content analyses. After developing the operational definitions of the second order codes (see Table 3), the researchers coded practice content–from questionnaires discarded as incomplete–that was not used in the present study and discussed coding discrepancies until agreement was reached.

They proceeded to independently analyze 18% of the sample (10 questionnaires) on which the Krippendorff’s alpha was calculated (Krippendorff, 1980) for the nominal values of the categories. Intercoder agreement exceeded the criterion set at 0.80 for all variables.

The statistical differences between male and female perceptions were calculated by SPSS statistical program. It was also used to calculate the percentages of the first-order codes. In addition, the same tool was used to analyze the accuracy of men’s and women’s perceptions of women’s representation in their respective police organizations. This variable is calculated as the difference between the perceived percentage of women in the organization minus the actual percentage of women, according to Eurostat data.

## Results

On the basis of the theoretical framework and especially in the research carried out by Natarajan (2008) and Silvestri and Tong (2020), concerning to the differentiated conditions faced by men and women in the workplaces, the results of the study are shown in four main themes as follows: (1) Perceived Representation (adequate representation and supporting female quotas); (2) The perceived nature of police work (police relevant competences, physical strength required and work-life balance); (3) Perceived organizational structure of law enforcement and the police subculture (male police subculture and perceived discrimination); and (4) Perceived stereotyping of female police officers (female specific skills, motivation, capacity and strength and female specific organizational barriers). Results are described in Table 4.

**TABLE 4:** Frequencies of second-order codes and Chi-square test.

					Chi-squared test			
	
					Value	df	Asymptotic Significance (2-sides)	Cramer’s V
			**Sex**		20.883^*a*^	1	0.000	0.611
	
			**Male**	**Female**				
	
Perceived adequate representation	Inadequate	Count	7	24				
		Expected count	15.5	15.5				
	adequate	Count	21	4				
		Expected count	12.5	12.5				
Supporting female quotas	Pro quotas	Count	2	20	24.257^*a*^	1	0.000	0,658
		Expected count	11	11				
	No pro quotas	Count	26	8				
		Expected count	17	17				
Police relevant competences	soft skills	Count	5	24	25.819^*a*^	1	0.000	0.679
		Expected count	14.5	14.5				
	soft and hard skills	Count	23	4				
		Expected count	13.5	13.5				
Physical strength	Required	Count	4	1	0.878^*b*^	1	0.349	0.188
		Expected count	25.5	25.5				
	No required	Count	24	27				
		Expected count	2.5	2.5				
Work-life balance	Yes	Count	25	17	6.095^*a*^	1	0.014	0.330
		Expected count	21	21				
	No	Count	3	11				
		Expected count	7	7				
Male police subculture	Male culture	Count	5	15	8.010^*a*^	2	0.018	0.378
		Expected count	10	10				
	Reflect of society	Count	9	4				
		Expected count	6.5	6.5				
	Old school	Count	14	9				
		Expected count	11.5	11.5				
Perceived discrimination	Bigger against women	Count	8	20	10.286^*a*^	1	0.001	0.429
		Expected count	14	14				
	no differences	Count	20	8				
		Expected count	14	14				
Female specific skills	Yes	Count	19	27	7.791^*a*^	1	0.005	0.373
		Expected count	23	23				
	No	Count	9	1				
		Expected count	5	5				
Motivation	Yes	Count	20	6	14,072^*a*^	1	0.000	0.501
		Expected count	13	13				
	No	Count	8	22				
		Expected count	15	15				
Capacity and strength	Yes	Count	10	2	6.788^*a*^	1	0.023	0.348
		Expected count	6	6				
	No	Count	18	26				
		Expected count	22	22				
Female specific organizational barriers	Yes	Count	7	26	26,635^*a*^	1	0.000	0.690
		Expected count	16.5	16.5				
	No	Count	21	2				
		Expected count	11.5	11.5				

*^*a*^0 cells (0.0%) have expected count less than 5.*

*^*b*^Continuity Correction in the χ^2^-test for 2 × 2 Tables.*

### Perceived Representation

#### Perceived Adequate Representation

As shown in Table 4, with regard to the general presence of women in their police organization, we observe that there is an over-estimation of the percentage of women, a fact that occurs to a much greater extent in the perception of male participants (*t* = 4.84; gl = 31.01; *p* < 0.000). Women have a much more accurate perception (*x* = −0.87; sd = 2.02) and, in some cases, underestimate the representation of policewomen, which is never the case for men (*x* = 5.4; sd = 6.49).

There is a very significant majority (85.7%) of women participants who perceive that there is not adequate representation of women police officers either in operational tasks, in command tasks, nor in front line tasks and the need to increase such representation, while only 25% of men participants clearly considers that women are not well represented (residual 8.5), χ^2^(1, *n* = 56) = 20.88, *p* < 0.000 Cramer’s *V* = 0.61.

#### Supporting Female Quotas

The existence of quotas to increase the presence of women in police organizations is a controversial issue supported by 39.2% of respondents. However, the gender difference is significant, with 71.4% of women in favor and only 7.1% of men. χ^2^(1, *n* = 56) = 24.26, *p* < 0.000 Cramer’s *V* = 0.66.

The advocates of quotas claim that they are necessary in certain posts and operational units, because of the existence of unequal treatment, which goes beyond the regulations, but which occurs in practice in a subtle way and leads to unequal opportunities with respect to men (residual = 9.00). They consider that this quota system can be an added value for limited circumstances such as “making women more visible” and “creating role models and networks,” Among the arguments against quotas, 14.28% of male participants believe that if quotas were to exist would lead to negative discrimination against men and that this type of measure would provoke resistance and the opposite of the desired effect.

### The Perceived Nature of Police Work

#### Police Relevant Competences

There are significant differences in the perception that men and women have over police-relevant competencies, as two sample Chi-square test showed χ^2^(1, *n* = 56) = 25.82, *p* < 0.000 Cramer’s *V* = 0.68.

A 85.7% of policewomen participants exclusively report on soft skills (residual = 9.50), such as “honesty,” “integrity,” “ethics,” “adequate emotional balance,” “flexibility,” “communication skills,” “planning,” “empathy,” or “cooperation skills,” 14.8% a combination of soft and hard competencies (residual = −9.50). In the case of the participating male police officers, the percentages are reversed to 17.9 and 82.1%, respectively.

#### Physical Strength Required

It is worth noticing that physical condition is hardly considered as a relevant competence and, although there are no significant differences between the perceptions of male and female participants, the percentage of men who cite it (14.28%) is higher than that of women (3.57%). χ^2^(1, *n* = 56) = 0.88, *p* > 0.05.

#### Work Life Balance

Respondents perceive difficulties in reconciling family life and work for policewomen, with men (89.3%) perceiving this perception to be higher than women (60.7%). Chi-square test (residual = 4.00) χ^2^(1, *n* = 56) = 6.09, *p* < 0.05 Cramer’s *V* = 0.33. While women talk about the “stress of personal life” and underline the idea of always having “double obligations and tasks,” men tend to consider the implications of supposed absenteeism due to pregnancy and children. Male participants, who perceive that women suffer specific barriers, give “greater weight to family planning,” especially in young women who are supposed to have children and will therefore leave work for a period of time (53.57%).

They add that “women cannot concentrate on work because they need to be in the care of the family” (7.14%), so they are “not promoted at the same time as men” (3.57%). They also give great weight to the fact that women “need to raise a family, have children, for which they choose office tasks, to reconcile schedules, pick up children, carry out domestic tasks and take care of the family” (39.28%). Only one man claims the need for work-life balance measures for men.

### Perceived Organizational Structure of Law Enforcement and the Police Subculture

#### Male Police Subculture

Female participants perceive the police culture as an unequal, masculine culture that establishes different criteria based on gender when assigning positions and evaluating performance (residual = 5.00) while male participants recognize to a greater extent an evolution and change in police culture thanks to the newer generations and the higher education they possess (residual = 2.50) χ^2^(2, *n* = 56) = 8.01, *p* < 0.05, Cramer’s *V* = 0.38).

Women perceive a predominantly male culture (53.6%) in which there are “power structures and influence structures,” a culture that establishes different functions and recognition and a “double standard when judging women and men,” with “women having to be harsh as men,” otherwise they are negatively evaluated.

The police organizations are seen as a paternalistic culture that upholds traditional cultural values, in which gender roles are reproduced (21.42%). According to the women surveyed, police organizations promote male culture and support and sustain men (42.85%), men are considered stronger and more capable (21.42%) and where “networks of influence and decision-making processes are in the exclusive hands of men” (32.15%). The stereotypes about women in their opinion translate into jokes, comments and specific attitudes toward women. They cite “obsolete prejudices,” “misinterpretation of erroneous roles and preconceptions,” “paternalism about women,” especially as “young people to be protected,” “inappropriate comments,” “rumors,” “prejudices about age,” the way in which “they have managed to ascend.” There is a “stereotype about women’s little or no ability to perform operative or leading positions” because they are “too emotional,” “overcome by their hormones,” “not assertive but hysterical,” “not active but nervous” (10.71%). Pressure to conform to male patterns of behavior would include penalties for attempting to exercise different leadership styles “even when they exceed your average performance” (17.85%). They also refer to the idea of “pregnancy as a limitation” because becoming pregnant and combining personal and family life favor the mistaken that “women do not have time,” “have other priorities” or “are not committed to work” (17.85%), that they are “perceived as inferior or as mothers” (10.71%). 32.1% of the female respondents believe that the change in police culture is already taking place and perceive a substantial difference between older and younger police officers. For them, the police subculture remains residual in the “old school.”

Regarding male participants, a percentage of 50% perceive that there is a difference between the “old school” of the oldest policemen and the current one, giving importance to “higher education” which, according to them, contributes to a “change in culture” (17.85%). They also state that “not all men are prejudiced” (17.85%) and recognize that in a masculine world “when you belong to a minority it is normal to be seen as strange” (14.28%). 32.1% reject the idea of the existence of a police subculture, considering that police organizations are a reflection of society. However, 10.71% of male respondents maintain that the police subculture exists, that it is still believed that “women are less valued in position of prestige,” that “only women who distance themselves from other women are valid to occupy positions of leadership.” To a lesser extent, they speak of the “vulnerability not only of women but also of fragile men who suffer from patriarchal culture” (3.57%) as well as “if they do not want to change jobs or location it seems that they cannot be excellent police officers” (3.57%).

#### Perceived Discrimination

The perception of a police subculture that discriminates against women is also evident in the analysis of the perceived prevalence of harassment, discrimination and violence against women. A two sample Chi-square test reveals that women perceive themselves as victims of more episodes of violence, harassment or discrimination in the workplace, and that men do not perceive this difference in treatment (residual = 6), χ^2^(1, *n* = 56) = 10.28, *p* < 0.001, Cramer’s *V* = 0.43.

Women perceive that they suffer horizontal discrimination (64.28%), feeling “confined to non-operational office tasks” and excluded from jobs “such as street work.” 28.57% also feel that they suffer vertical discrimination compared to their colleagues, being “assigned to insignificant,” “non-prestigious,” “second-level” jobs, relegated to positions that put them in an “unfavorable position for promotion.” They also claim (7.14%) that they occupy leadership positions “only when these have been discarded by men.” Women also perceive pay discrimination because they “receive less rewards, recognition and in some cases less pay,” and isolation–“men sometimes openly refuse to work with women” or “colleagues do not share information or collaborate” with them (10.71%). A majority of women say they are subject to more scrutiny than their male colleagues caused by “over-generalization of women’s failures”–“demonstrating their worth,” “have demonstrated skills and validity for the position.” To a lesser extent, at 14.28%, they say that policewomen suffer psychological harassment, referring to sexist behavior or attitudes, comments about their “physical attributes rather than their skills,” jokes–“macho comments encouraged or consented to by superiors”–rumors about “intimate relationships with superiors,” “membership of a political party or group of influence.” Lastly, a percentage of 21.42% of the women participants do not perceive more discrimination toward women and link the episodes that might exist to the rank of the people and to rural areas.

Men perceive to a much lesser extent (28.6%) that women suffer more episodes of violence, harassment or discrimination at work, referring to the fact that “laws already regulate these matters,” and that they are events that could have occurred in the past, but that at the present time they are not common phenomena. 7.14% recognize vertical discrimination– “they occupy leadership positions only when these have been discarded by men”–but, above all, they perceive that the cases that happen “may be related to jokes,” (21.42%) “sexist comments” (14.28%), “rumors about intimate relationships” (7.14%), “greater attention” (3.57%). With regard to the complaints expressed by women about this type of harassment, sometimes attribute it to “the very behavior of the woman who does not confront such behaviors” (3.57%), to the existence of a “typical female victim profile” (3.57%), to the “vulnerability of women” which results in them suffering more (3.57%) or to “human nature” (3.57%).

On the other hand, the perception of discrimination suffered by women is related to support for the existence of quotas as an affirmative action measure. 71.4% of participants who perceived greater discrimination against women were in favor of implementing quotas (residual = 9.00). χ^2^(1, *n* = 56) 24.26, *p* < 0.000, Cramer’s *V* = 0.66.

### Perceived Stereotyping of Female Police Officers

#### Female Specific Skills

There is a different perception of the existence of specific skills that would characterize female police officers in the work environment. χ^2^(1, *n* = 56) = 7.79, *p* < 0.005 Cramer’s *V* = 0.37.

Women mainly perceive the existence of values such as integrity, care of reputation, as well as social and management or organizational skills (96.4%). Men, approximately a third of the participants, consider that women and men do not differ in specific language, although there is a high percentage of participants (67.9%) who associate women with “greater empathy,” “emotion,” “ability to communicate” and who place them directly as “better in tasks of attention to citizens” and specifically as investigators of “crimes that affect women,” “minors,” “elderly people” as well as “vulnerable victims.”

#### Motivation

The two-sample Chi-square test reveals the importance that men (71.4%) attach to motivational factors in explaining women’s career development vis-à-vis their female peers χ^2^(1, *n* = 56) = 14.07, *p* < 0.000 Cramer’s *V* = 0.50.

The highest percentage of male participants (60%), consider that women “prefer non-operational positions,” “they manage to place themselves before men in administrative tasks” or “women are not interested in working in the street.” 46.4 % consider that there is a lack of motivation and ambition among women–that they do not want “to be forced to move up,” that they do not want to “change their professional destination,” that they put their personal life first–“women do not have time,” “have other priorities” or “are not committed to work”–even though “single women can choose operational tasks.”

#### Capacity and Strenght

To a lesser extent, men also question the capacity and strength of their female colleagues. 35.7% of men say that women have some demerit compared to men, compared to 7.1% of women. χ^2^(1, *n* = 56) = 6.79, *p* < 0.01 Cramer’s *V* = 0.35.

Men consider women’s vulnerability exempts them from certain posts–“they are good at administrative tasks, but not in the street where they cannot defend themselves” or “they are more vulnerable and must be protected” (7.14%), that “they cannot imagine themselves in special units.” They also refer to biological factors that make them less capable for the job–“they have special biological needs such as going to the toilet or not being able to stand night shifts or bad weather conditions,” “they have less physical skills and… they always look for work-life balance measures”. Some components of hostile sexism also appear in the answers given by men: “they are emotional,” that “they take advantage of the situation through seduction and manipulation, sexualising the work environment, that they do not change,” “two women together are not compatible”; that “in certain positions they can create problems.”

#### Female Specific Organizational Barriers

In response to the lack of motivation and capacity attributed to women, the vast majority of female police respondents (92.9%) cited organizational factors to explain their low presence and difficulties in career development. These factors are only considered by 25% of male respondents. χ^2^(1, *n* = 56) = 26.63, *p* < 0.000 Cramer’s *V* = 0.69.

A 46.42% of the women mentioned non-transparent selection and evaluation processes. Most of them consider that in police organizations, male characteristics prevail in the recognition of merit–“even when they are above a male candidate in experience, responsibility and validity, based on the argument that a woman does not have enough experience or leadership skills”. They also believe that promotion processes are not transparent and that they are not aware of existing opportunities. They perceive that they lack support (22.5%) and that they are victims in the processes of “hidden and silent maneuvers.” 32.14% hold the culture of the organization directly responsible for their situation–“masculine culture itself always penalizes women in a bubbling and unofficial way.” This weight of culture and prevalence is also recognized by men, although in no case do they mention the selective processes or the lack of support for women.

## Discussion

Overall, our results, based on the data provided by the senior male police officers participating in our study, exhibit considerable blindness to the inequalities highlighted by their female counterparts. The following is a discussion of the findings, based on the four main themes described in the results: Representation, the nature of police work, organizational structure of law enforcement and the police subculture and stereotyping of female police officers.

### Representation Perceived by Police Executive Leaders

One of the most striking manifestations of this mirage is that they claim that women’s representation is adequate and that no organizational measures need to be taken to increase it. This result is consistent with others found in the literature and which agree that, when the presence of women is overestimated, as in the case demonstrated here, positive action measures are negatively valued (Dolan, 2014). Lombardo and Mergaert (2013) find that denial of the need for gender change is a frequent point of resistance to achieving a balanced workforce. One of the ways in which this resistance manifests itself is in disbelief of gender data.

Moreover, our results are also in line with previous studies that found that women had more positive attitudes toward affirmative action than men had (Konrad and Linnehan, 1995; Beaton and Tougas, 2001; Golden et al., 2001; Konrad and Hartmann, 2001; Fleischmann and Burgmer, 2020).

### The Nature of Police Work Perceived by Police Executive Leaders

In relation to the issues and controversy raised about the entry of women into police forces, there is a recurrent idea of the vulnerability of women in operational positions or special units (Rabe-Hemp, 2008), despite studies showing otherwise (Poole and Pogrebin, 1988; Worden, 1993). However, the perceptions of essential policing skills of both the men and women participating in this study do not prioritize physical condition as pointed out by Lanier (1996) and Natarajan (2008). This paradoxical result is already a classic in research on policewoman and other traditionally male professions (Román et al., 2013; Natarajan, 2008). It has been a question of debate whether the physical tests prior to selection discriminate positively against women by requiring other less demanding scores than men, or whether from another perspective, they aim to eliminate indirect discrimination. Several studies highlight that the requirements to pass certain physical tests must be reviewed, since it has been demonstrated that the requested skills are rarely used in the street (Birzer and Craig, 1996; Rabe-Hemp, 2008), so that the debate can move on toward a reflection on the suitability of physical tests to evaluate police competencies, fundamentally in operational tasks and what kind of orientation these physical capabilities have: muscle power and strength–namely masculine–or other skills such as agility, flexibility, or physical endurance.

Based on the research results it can be concluded that female participants express as essential the qualities of honesty, reputation, social skills, openness to change and communication i.e., those that coincide with qualities of transformational leadership and are identified as soft competencies (more people-oriented). Male participants perceive as more relevant the ability to integrate and lead teams, to make decisions and to have professional competence. As Kakar (2002) highlights, there seem to be stereotyped perceptions of women’s “soft” skills, which are not considered a value, contrary to the various studies by Ayman et al. (2009) and Vinkenburg et al. (2011), which demonstrate that women have repeatedly been shown to be successful at implementing a transformational style of leadership and that they are capable of bringing about effective organizational change, skills that are considered necessary and desirable in police services. The perception of men tends to consider these skills as of lesser value, less recognized and less adjusted to the dominant profile, cataloging women as emotional, with little capacity to take risks and mostly oriented toward people.

There appears to be role incongruence in women, as stated by Mainiero and Sullivan (2005). Male participants affirm that family, children, and domestic obligations are something that policewomen “want, seek, need,” without, on the contrary, observing, except in minimal exceptions, that these same men project themselves as fathers, husbands or those responsible for domestic management. According to the authors, men within organizations kept their personal lives and work separate, being able to do so as their partners normally dealt with the delicate game of balancing work and personal issues.

Cáceres et al. (2012) argue that maternity leave, if considered ineffective, can lead to indirect discrimination in promotions. This is assumed by women when they perceive subtle criteria and hidden maneuvers of discrimination. Men perceive such penalization as inevitable, although they consider that women have the same opportunities and rights as men, therefore, except for a very low percentage, they do not perceive specific barriers in women by gender, but they consider that the common barriers in men and women for career development are excessive youth, lack of experience, having more training and not occupying relevant or prestigious positions for promotion.

### Organizational Structure of Law Enforcement and the Police Subculture Perceived by Police Executive Leaders

According the results, the perception of the incidence of harassment, violence and discrimination against women also differs between men and police in the study presented here. Although women perceive sexist discriminatory behavior, jokes, comments about their physical appearance, their ways of getting promoted, coming mainly from the most veteran policemen and, in some cases, encouraged or consented by the bosses themselves, policemen do not believe that these events differ quantitatively from those suffered by men. Furthermore, they attribute the positive cases to the fact that they receive more attention, that they are a minority, that they do not confront comments or attitudes, that they are a type vulnerable profile or that they even sexualize the work environment. The blaming of the victimized women using these types of arguments also maintains the illusion of equality and justice and has been explained by the theory of the mixed content of stereotypes (Fiske et al., 2002) and that of ambivalent sexism (Glick and Fiske, 1996).

According to our results, men do perceive a higher incidence of discrimination against women, as do women, support the implementation of quotas. This finding has been confirmed in several studies (Kravitz and Klineberg, 2000; Aberson, 2007) where people who perceive discrimination against a certain group are more likely to support affirmative action measures toward it. Although this result has not always been confirmed at the organizational level (Fleischmann and Burgmer, 2020).

The mirage of equality that blinds experienced policemen in this studio is no exception, not even a rarity in our society (Lombardo and Mergaert, 2013). As in other social areas, the struggle for real equality must overcome the veil woven by the visibility of women in previously forbidden spaces, the general acceptance of gender issues in the agendas of administrations, the use of language, the media presence and collective thinking, among others (García Prince, 2005; Lagarde, 2013). In the case of the police organizations, the presence of token women (Kanter, 1977), who enjoy greater visibility and who serve as a guarantee of equality among all–regardless of the real access they have to power–, the acceptance and inclusion of equal rights regulations–already achieved in past stages (Brown, 1997) at least, on paper-, the use of the image of policewomen officers publicly in communication campaigns (Davies and Hartono, 2015), the incorporation of new generations of police officers, who are better trained and have democratic and egalitarian values, can be the threads with which the blindfold of inequality is woven.

As stated by Ramos et al. (2003) and Cuadrado et al. (2012), women perceive obstacles such as the so-called “glass ceiling” or vertical promotion, but they identify above all with the “glass wall,” which determines and fits them to perform only certain areas of work. In our study, male participants themselves recognize this glass wall, but as a shared belief, a basic assumption of policing culture based on women not only possessing more managerial skills, but also wanting to occupy those administrative positions rather than for street positions. Contrary to what Natarajan (2008) argues, the women in this study perceive that segregation into administrative and non-operational tasks hinders their promotion and does not enable them to achieve the same status as men within the police organization. The policewomen in this study agree with the conclusions reached more than two decades ago by French and Waugh (1998) about the power that segregation has in perpetuating the stereotype of the weaker sex in police organizations. Indeed, women referring to the lack of opportunities to access relevant and prestigious positions, and certain units, the so-called special units, about which they say that in some countries there is a rule that prohibits women’s access to them, or limits them, or in other cases, even though there is no regulation against women’s entry, the reality is that they do not have access to these units, or they only have access to limited positions within them, such as administrative positions.

### Stereotyping of Female Police Perceived by Police Executive Leaders

In our study, both men and women ratify the term “Homosocial Reproduction” coined by Kanter (1977), as the process by which relevant decisions are based on positively evaluating and promoting those with similar characteristics, with the caveat that women participants consider men to support other men, and male participants affirm that support is given to those who are in relevant positions and immersed in certain networks, considering as relevant those operational, not management positions.

Metz and Kulic (2008) emphasized that strong organizational cultures create a strong consensus and cohesion, a kind of dysfunctional culture that causes people who are different in appearance, attitudes and behaviors to be excluded and prevented from advancing. Dysfunctional cultures serve to protect the *status quo*, maintain exclusivity, so they will resist any internal change and encourage their organization to be inclusive (Conceição and Altman, 2011). In addition to the cultural artifacts reported by the female police officers interviewed, which exclude them from decision-making processes and positions of power, the police culture, according to our results, continues to uphold gender stereotypes.

The lack of motivation is also, for the male police in our study, the main reason why women do not reach relevant leadership positions. The results show that male participants perceive that woman have little time, no ambition, do not want or take longer to ascend, and attribute less experience to them. On the other hand, the hypothesis that women are still not represented in management positions because it is a male perspective that determines the selection criteria (Barberá et al., 2011; Silvestri, 2013), is supported by the perceptions of the participating women. Men, while agreeing on the inadequacy of selection systems, do not perceive that it is due to a male majority in decision making panels.

A high percentage of women associate social stereotypes with professional stereotypes in police organization. These stereotypes are based on the devaluation of their physical abilities, especially for their appearance, behavior and leadership style. They also cite paternalistic attitudes that translate into different treatment, differences in status and power and the idea, with a lower percentage, that emotion and hormones dominate them, as well as the role of women as mothers. Men, in a high percentage, believe that it is women’s own decision or personal choice not to perform operational tasks. They also argue biological reasons for their non-integration or effectiveness in certain non-administrative functions. They also perceive fairly uniformly that it is their personal needs to raise a family, have children and take care of household chores that diminish their ability or ability to concentrate or commit to work. A small percentage of men attribute to internal factors, within the organization itself, the lower value that women have in certain prestigious positions and in the recognition of their achievements.

For women executive leaders, however, the factors to which they attribute the difficulties they experience in their incorporation and promotion are linked to the organization itself; its culture, human resources processes, their lack of participation in decision-making processes and the lack of internal support.

## Conclusion

The aim of this research was to analyze what barriers executive leaders perceive as hindering the recruitment and career development of policewomen in Europe, using a purposive sampling of 56 police officers from 26 European countries. The results obtained show four major themes: (1) the nature of police work; (2) the organizational structure of law enforcement; (3) the police subculture and stereotyping of female police officers and (4) women’s representation. The first three major themes that emerged from this research are in line with previous studies described in the literature review (i.e., Natarajan, 2008; Silvestri and Tong, 2020). The fourth area–women’s representation–is consistent with previous literature on resistance to organizational change involving gender mainstreaming (Agócs, 1997), but is novel in police organizations.

Based on the results, it is possible to conclude that there are significant differences in the perceptions of male and female police executive leaders with regard to access, career development and workplace conditions faced by policewomen and men. According to the results, the mirage of equality, dominant in the view of male police officers, highlights a major barrier to achieving real equality, both horizontally and vertically.

The responses of the participants in this study are polarized into the two models of assimilation of women into the police force proposed by Heidensohn (1998). Although male police officers hide behind the existence of norms that ensure equality to deny inequalities, their perception of the organization leads them to defend a “gendered” assimilation model that limits the presence of women to certain tasks and positions. Women, however, defend the integrated model, which confers them with the same rights to be part of the police organization and to aspire to the position they want to occupy, just like men.

Frances Heidensohn (1998) suggested that the problem of inequality persists globally because “it has deep roots and powerful protectors.” She proposed the use of strategies at three levels: nationally (state or federal), service or agency, individual.

Following her recommendations, at a national level, countries should continue with the national development of European legislation on equality and non-discrimination. Our results show serious difficulties in balancing family and professional roles for women where women feel overloaded and men feel it is an impossibility to achieve this balance while maintaining a sufficiently adequate professional performance. Therefore, conciliation policies that grant non-transferable parental leave between men and women can help to modify the cultural values of police organizations as well as to reduce the overload experienced by women.

With regards to the second strategic level of service or agency, there should be a critical review within police organizations, of how structures policies, processes, protocols, and practices are having a different impact on men and women, both in the organization itself, as well as on citizens and external personnel. Female executive leaders’ perceived barriers in this study associated with selection, promotion and communication processes, as well as their absence from formal and informal structures of influence, suggest the need to introduce professionalized human resource management systems in which women are involved. Correct job descriptions and competency-based evaluation require an effort to train those responsible for selective and promotion processes. Encouraging the existence of women’s networks and mentoring programs could reduce the isolation of women within police organizations.

The most relevant result of our study indicates that the intervention in the third strategic level–the individual–is not only essential but its omission puts the previous interventions at risk. The male executive leaders participating in our study show explicit resistance to the introduction of a gender perspective, as in other types of institutions (Lombardo and Mergaert, 2013). Our results show that they do not believe it is necessary to introduce gender changes. The manifestations of this resistance take the form of disbelief of gender data, the mirage of equality, or the defense of traditional gender roles as natural. Therefore, we argue that the results of this study underscore the need to include, in the strategic level of the individual, the development of new masculinities that improve the capacity to recognize the privileged situation in which men live and correct the attribution errors they make regarding their female colleagues. As Fleischmann and Burgmer (2020) suggest, deep reflection and the development of abstract thinking about existing inequities is the path to increase support for gender mainstreaming.

### Limitations of the Study

Due to the number of participants, this research should be considered a pilot study with a limited capacity for generalization. The difficulty in accessing senior police officers in the various organizations has been notable. It would be desirable to expand the number of interviews or, based on these results, design another information collection strategy.

The sample size has probably also not allowed for the collection of information on forms of intersectional discrimination linked to, for example, sexual orientation, age, ethnicity, or religious beliefs.

Deliberately, the vision collected has been that of people with wide-ranging experience in the organization. This is also a partial approach. In the future, it would be advisable to also access people from other ranges and levels of police experience.

One of the limitations of this study refers to the lack of access to objective data on percentages of women in leadership positions in some European countries. The following is information that was unavailable at the time of study but would have been relevant: the exact number of candidates in each country, men and women, in both the admission and promotion processes and the tests by which they were excluded. Likewise, the lack of homogeneity in the processes of entry and promotion, the existence of different structures, military and/or civilian, and therefore also the different denominations to the same or different ranks, make it very difficult to establish the real limitations in the selection processes or the criteria that allow free access, internal promotion to the different scales, as well as the importance given to merit and seniority, and which are the exact criteria that are measured in performance evaluations or in personal interviews. Given the organizational and social particularities from which the participants in the study responded, it would be desirable to make specific studies in each of the organizations and countries.

## Data Availability Statement

The raw data supporting the conclusions of this article will be made available by the authors, without undue reservation.

## Ethics Statement

Ethical review and approval was not required for the study on human participants in accordance with the local legislation and institutional requirements. The patients/participants provided their written informed consent to participate in this study.

## Author Contributions

All authors listed have made a substantial, direct and intellectual contribution to the work, and approved it for publication.

## Conflict of Interest

The authors declare that the research was conducted in the absence of any commercial or financial relationships that could be construed as a potential conflict of interest.

## Publisher’s Note

All claims expressed in this article are solely those of the authors and do not necessarily represent those of their affiliated organizations, or those of the publisher, the editors and the reviewers. Any product that may be evaluated in this article, or claim that may be made by its manufacturer, is not guaranteed or endorsed by the publisher.
