# Supplementary material for: Is It Because You Don’t Want to? A Content Analysis of Police Executive Leaders’ Perceptions of Policewomen’s Careers in Europe
Source: Front Psychol. 2021 Sep 27;12:713696. doi: 10.3389/fpsyg.2021.713696 (PMC8503680; doi:10.3389/fpsyg.2021.713696)
Supplement: Supplementary file 1 [file Data_Sheet_1.pdf]

ANNEX I. Interview Guide

Country Gender

Rank Post

Years of Police Experience

Interview Guide Questions	Contribution to Main Research Questions
1.- How is the police structure in your country? (Civil or military character) (One to more Police Forces)	3) How they perceive the culture, organizational structure, leadership style, human resources processes and equality policies?
2.- How is the hierarchical structure (scales and categories)?	3) How they perceive the culture, organizational structure, leadership style, human resources processes and equality policies?
3.-What are the access requirements?	3) How they perceive the culture, organizational structure, leadership style, human resources processes and equality policies?
4.-These requirements are the same for men and women. What do you think about?	2) Do they perceive that women differ from men in policing?
5.-What is the more or less percentage of men and women in the police structure.	2) Do they perceive that women differ from men in policing?
6.-How is the promotion system? (Requirements, different systems of promotion)	3) How they perceive the culture, organizational structure, leadership style, human resources processes and equality policies?
7.-How many years ago did women join the Police Organization?	1) From police executive leader’s perception, how desirable is to develop measures to achieve gender parity in the staffing structures?
8.-How many women occupy positions that are considered managerial or high-level?	1) From police executive leader’s perception, how desirable is to develop measures to achieve gender parity in the staffing structures?
9.-Are there any quotas in your organization (seats reserved for women) or specific positions to be filled by women? What do you think about this?	1) From police executive leader’s perception, how desirable is to develop measures to achieve gender parity in the staffing structures?
10.-Are there any specific regulations regarding equality measures in your organization (protocols, positive actions regarding sexual harassment, labor, conciliation measures, gender violence)?	3) How they perceive the culture, organizational structure, leadership style, human resources processes and equality policies?
11.-Is there an equality office in the structure?	3) How they perceive the culture, organizational structure, leadership style, human resources processes and equality policies?

12.-Is there any mentoring program or women's networks?	3) How they perceive the culture, organizational structure, leadership style, human resources processes and equality policies?
13.- Do you believe that women tend to choose or prefer certain tasks or positions within police organizations?	2) Do they perceive that women differ from men in policing?
14.-Do you consider that women differ in performance in some kind of task, positively or negatively (operational tasks, special groups, management groups, etc.)	2) Do they perceive that women differ from men in policing?
15.-What skills or characteristics do you consider most relevant for being a police officer in your country?	2) Do they perceive that women differ from men in policing?
16.-What do you think is the type of leadership exercised by managers in your organization?	3) How they perceive the culture, organizational structure, leadership style, human resources processes and equality policies?
17.-Do you think that women have specific skills, different from men, when they hold managerial positions?	2) Do they perceive that women differ from men in policing?
18.- What you think are the prejudices, barriers or stereotypes a woman suffers in career development?	2) Do they perceive that women differ from men in policing?
19.- What are the barriers, obstacles or prejudices you have had to face in your career development or promotion?	2) Do they perceive that women differ from men in policing?
20.-Do you think women suffer more episodes of violence, harassment or discrimination in the workplace? If yes, what is it based on?	2) Do they perceive that women differ from men in policing?
21.-Do you believe that women are well represented in managerial positions or decision-making processes? Briefly explain your answer	1) From police executive leader’s perception, how desirable is to develop measures to achieve gender parity in the staffing structures?
22.-Do you think a policy is needed to increase the number of women in managerial positions? Briefly explain your answer	1) From police executive leader’s perception, how desirable is to develop measures to achieve gender parity in the staffing structures?
23.-What is your professional experience with policewomen, companions, subordinates or bosses	2) Do they perceive that women differ from men in policing?
